# Increased systemic and brain cytokine production and neuroinflammation by endotoxin following ethanol treatment

**DOI:** 10.1186/1742-2094-5-10

**Published:** 2008-03-18

**Authors:** Liya Qin, Jun He, Richard N Hanes, Olivera Pluzarev, Jau-Shyong Hong, Fulton T Crews

**Affiliations:** 1 Bowles Center for Alcohol Studies, School of Medicine, CB#7178 UNC-CH, Chapel Hill, NC 27599, USA; 2 Neuropharmacology Section, NIEHS, RTP, NC 27709, USA

## Abstract

**Background:**

Cytokines and alcohol share a common modulation of inflammation and hormones as well as being implicated in multiple diseases, but the mechanisms are poorly understood. The purpose of this study was to investigate the interaction of liver, serum and brain cytokines as well as whether ethanol would potentiate endotoxin (Lipopolysaccharide, LPS) responses once ethanol had cleared.

**Methods:**

Male C57BL/6J mice were treated intragastrically with water (control) or ethanol (5 g/kg, i.g., 25% ethanol, w/v), with volumes matched, for 1 day or daily for 10 days. Mice were then injected intraperitoneally with saline (control) or LPS (3 mg/kg, i.p.) in saline 24 hrs after the last dose of ethanol. Gene expression and protein synthesis of proinflammatory cytokines and anti-inflammatory cytokine, oxidative enzymes, microglial activation and inhibition of neurogenesis were examined using real-time PCR, ELISA, and immunohistochemistry.

**Results:**

LPS increased proinflammatory cytokines (TNF***α***, MCP-1, IL-1***β***) several fold in liver, brain and serum at 1 hr. Ethanol is known to increase liver cytokines and alter the risk of multiple chronic diseases. Ten daily doses of ethanol increased brain and liver TNF***α***, and pretreatment with ethanol potentiated LPS-induced increases in TNF***α***, MCP-1, IL-1***β ***in liver, serum and brain. Proinflammatory cytokine levels in liver and serum returned to basal levels within a day, whereas brain proinflammatory cytokines remained elevated for long periods. IL-10, an anti-inflammatory cytokine, is reduced in brain by ethanol and LPS, while brain proinflammatory cytokines remain increased, whereas liver IL-10 is increased when proinflammatory cytokines have returned to control levels. Activation of brain microglia indicated by morphological changes, reduced neurogenesis and increased brain expression of COX-2 and gp91^phox ^NADPH oxidase subunit mRNA were found in the 10 daily doses of ethanol-pretreated LPS group.

**Conclusion:**

Acute increases in serum cytokines induce long lasting increases in brain proinflammatory cytokines. Ten daily doses of ethanol exposure results in persistent alterations of cytokines and significantly increases the magnitude and duration of central and peripheral proinflammatory cytokines and microglial activation. Ethanol induced differential anti-inflammatory cytokine IL-10 responses in liver and brain could cause long lasting disruption of cytokine cascades that could contribute to protection or increased risk of multiple chronic diseases.

## Background

Cytokines have been increasingly recognized as factors much like hormones regulating homeostatic responses among multiple tissues. One role of cytokines is to modulate inflammatory responses by shifting from Th1, proinflammatory cascades, to Th2, anti-inflammatory healing cascades [1]. Tumor necrosis factor alpha (TNF***α***), a key proinflammatory cytokine, induces the secretion of other cytokines and enzymes in various cells and tissues. Inflammation and proinflammatory cytokine cascades have been implicated in a variety of diseases affecting heart, lung, and liver, as well as the central nervous system. The gram-negative bacteria endotoxin, lipopolysaccharide (LPS), activates proinflammatory cytokine cascades through plasma membrane proteins, e.g. the toll-like receptor 4 (TLR4) and CD14, which leads to the production of TNF***α ***and other proinflammatory cytokines. In addition, TNF***α ***and other cytokines regulate the release of adrenal and other hormones, and also affect central nervous system function. Although LPS has been extensively used to study proinflammatory effects on peripheral tissue/organs, the influence of systemic inflammation on brain cytokines is not clear. Recently, we found that systemic LPS administration induced TNF***α ***production in liver, serum and brain within one hour [2]. These studies indicated that TNF***α ***from serum rapidly activates a proinflammatory cascade in brain. In the present study we extend these findings to varied doses of LPS that range from low to maximal cytokine responses [2]. Further we measured the proinflammatory cytokines MCP-1 and IL-1β, since MCP-1 has been found to be increased in the brains of alcoholic humans [3] and IL-1β is a key proinflammatory cytokine associated with neurodegeneration [4]. In addition, we measured IL-10, a Th2 prototype also known as human cytokine synthesis inhibitory factor, which down-regulates the expression of proinflammatory Th1 cytokines, MHC class II Ags, and costimulatory molecules on macrophages. In the present study we determined the effects of ethanol and LPS on these multiple cytokines in order to better understand the relationship among liver, serum and brain cytokine synthesis.

To gain insight into the impact of ethanol-LPS on brain we measured the proliferation and differentiation of neuroprogenetors in hippocampal dentate gyrus. These progenitors have been implicated in regulating mood and certain cognitive abilities. Hippocampal neuroprogenetors are inhibited by both ethanol [5] and LPS [6]. Changes in brain progenitors could contribute to the degeneration associated with prolonged neuroinflammation and alcohol abuse.

Alcohol (ethanol) is a common dietary constituent that impacts health. Although moderate alcohol consumption has a protective effect on heart disease and appears to also have other health benefits, heavy drinking increases mortality by escalating the risk of multi-system diseases in peripheral organs as well as psychiatric and neurological disorders in the central nervous system [7]. The mechanisms of alcohol induced benefits or increased risk of morbidity are not well understood. Generally, alcohol abuse is associated with disruption of immune defenses against infections, increased incidence of bacterial pneumonias, higher rates of chronic hepatitis C infection, and increased susceptibility to HIV infection [7-10]. In humans, chronic alcohol consumption is associated with increases in serum proinflammatory cytokines including TNFα, IL-1β, and other cytokines [11,12]. Monocytes isolated from the blood of alcoholics produce greater amounts of TNFα spontaneously and in response to endotoxin challenge [13]. Chronic alcohol consuming pregnant women and their fetus show elevated levels of blood cytokines including TNFα, IL-1***β ***and IL-6 as determined by using cord blood [14]. Interestingly, studies have suggested that TNFα through regulation of leptin formation contributes to craving for alcohol [15]. Ethanol induced alterations in cytokines might contribute to both the health benefits and increased risk for poor health associated with alcohol consumption. Although acute alcohol can directly inhibit endotoxin cytokine responses *in vitro *[1], the effects of chronic ethanol exposure have not been systematically studied. To avoid the acute direct effect of ethanol, cytokines were measured 24 hours after ethanol treatment to ensure the absence of ethanol before measuring cytokines. We report here that ethanol exposure induces liver, serum and brain cytokines and markedly increases LPS-induced cytokine responses. Liver and systemic cytokine responses decline within one week, associated with an increase in IL-10, an anti-inflammatory cytokine, whereas in brain a long lasting increase in proinflammatory cytokines (TNF***α***, IL-1***β ***and MCP-1) occurs while IL-10 is reduced. These findings on systemic proinflammatory cytokines, anti-inflammatory cytokines and long lasting brain induction of proinflammatory cytokines could have an important impact on physical and mental health.

## Materials and Methods

### Animals

Eight-week old male (20–22 g) C57BL/6J mice were purchased from Jackson Laboratories (Bar Harbor, Maine). Animals were randomly assigned to different groups and treated according to each group protocol. All protocols in this study were approved by the Institutional Animal Care and Use Committee and were in accordance with the National Institute of Health regulations for the care and use of animals in research.

### Reagents

Lipopolysaccharide (LPS, strain O111:B4) was purchased from Calbiochem (San Diego, CA). Rabbit anti-Iba1 antibody was purchased from Wako Pure Chemical Industries, Ltd. (1–2Doshomachi 3-Chome Chuo-ku Osaka 540–8605, Japan). Anti-mouse PCNA was purchased from DAKO Corporation (Carpinteria, CA) and anti-goat doublecortin was purchased from Santa Cruz Biotechnology (Santa Cruz, CA). TNFα, IL-1***β***, MCP-1 and IL-10 ELISA kits were purchased from R & D Systems Inc. (Minneapolis, MN). All other reagents came from Sigma Chemical Co. (St. Louis, MO).

### Animal treatments

Male C57BL/6J mice were treated intragastrically with water (control) or ethanol (5 g/kg, i.g., 25% ethanol w/v), with volumes matched, for one day or daily for 10 days. Blood alcohol level at 1 hr after a single dose of ethanol (5 g/kg, i.g.) was 310 mg/dl ± 8.9 (w/v, n = 12). Levels of 10 daily doses of ethanol were measured at 1 hr after the first dose of ethanol (5 g/kg, i.g.) and 1 hr after the ninth dose of ethanol, which are 304 mg/dl ± 12 (w/v, n = 12) and 316 mg/dl, ± 11 (w/v, n = 12), respectively. These blood ethanol levels are high and considered to model binge drinking [16]. Mice were then injected intraperitoneally with saline (control) or LPS (3 mg/kg, i.p.) in saline 24 hrs after the last dose of ethanol. Mice were sacrificed at indicated time points (1 hour and 1 week following LPS injection), and their livers, brains and sera were used for either morphological or biochemical (mRNA and protein) analyses. Observation of potential toxicity of these treatments revealed that ethanol treated mice did not show significant signs of toxicity. Grooming declined initially. Although 10 day ethanol treatment groups showed a trend to lower body weights, body weight was not significantly different from controls. Procedures using laboratory animals were in accordance with the National Institutes of Health guidelines for the use of live animals and approved by IUCAC boards.

### Immunohistochemistry

Brains were fixed and processed for immunostaining as described previously [17]. Microglia were stained with rabbit anti-Iba1 antibody. Immature and newborn neurons were stained with either anti-mouse proliferating cell nuclear antigen (PCNA) or anti-goat doublecortin antibodies. Immunostaining was visualized by using nickel-enhanced 3,3'-diaminobenzidinne (DAB) as a chromagen.

### Real-time PCR analysis

Total RNA was extracted from the mouse brain and liver samples treated with ethanol, LPS, ETOH-LPS or saline at the indicated time points and reverse transcribed as described previously [17]. The primer sequences used in this study were as follows: TNFα, 5'-GAC CCT CAC ACT CAG ATC ATC TTC T-3' (forward) and 5'-CCT CCA CTT GGT GGT TTG CT-3' (reverse); IL-1β, 5'-CTG GTG TGT GAC GTT CCC ATT A-3' (forward) and 5'-CCG ACA GCA CGA GGC TTT-3' (reverse); MCP-1, 5'-ACT GAA GCC AGC TCT CTC TTC CTC-3' (forward) and 5'-ACT GAA GCC AGC TCT CTC TTC CTC-3' (reverse); COX-2, 5'-GCT GGC CTG GTA CTC AGT AGG TT-3' (forward) and 5'-CGA GGC CAC TGA TAC CTA TTG C-3'(reverse);gp91^phox^, 5'-CAG GAG TTC CAA GAT GCC TG-3' (forward) and 5'-GAT TGG CCT GAG ATT CAT CC-3' (reverse); p67^phox^, 5'-CAG CCA GCT TCG GAA CAT G-3' (forward) and 5'-GAC AGT ACC AGG ATT ACA TC-3' (reverse); DBI, 5'-GCC AGT ATG TCT CAG GCT GA-3' (forward) and 5'-AGG CAT TAT GTC CTC ACA GG-3' (reverse); IL-10, 5'-GGT TGC CAA GCC TTA TCG GA-3' (forward) and 5'-ACC TGC TCC ACT GCC TTG CT-3' (reverse); β-actin, 5'-GTA TGA CTC CAC TCA CGG CAA A-3' (forward) and 5'-GGT CTC GCT CCT GGA AGA TG-3' (reverse). The SYBR green PCR master mix (Applied Biosystems, Foster City, CA) was used for real-time PCR analysis. The relative differences in expression between groups were expressed using cycle time (Ct) values normalized with β-actin, and relative differences between control and treatment groups were calculated and expressed as relative increases setting control as 100%.

Beta-actin is an abundant mRNA commonly used to normalize less abundant mRNA [18]. To ensure that treatments did not alter beta-actin levels the Ctvalues of the beta-actin were compared between groups. For example, for Table 1, a comparison of control to the 10 daily doses of EtOH group the average C_T _value was 18.75 ± 0.14 and 18.87 ± 0.06, p = 0.48, n = 5, respectively. No significant difference was found in any treatment groups for beta-actin. Therefore, we used beta-actin to normalize mRNA levels.

**Table 1:** Ten daily doses of ethanol and LPS induced changes in gene expression in brain and liver

Gene	Control	ETOH	LPS	ETOH + LPS
**Brain**				
TNF***α***	100 ± 19	362 ± 73**	9147 ± 3080*	9229 ± 1464*
MCP-1	100 ± 5	130 ± 7**	618 ± 156**	559 ± 186*
IL-1***β***	100 ± 9	133 ± 20	4172 ± 1115**	3962 ± 651*
COX-2	100 ± 10	82 ± 8	459 ± 43**	487 ± 72**
gp91^phox^	100 ± 11	194 ± 19**	155 ± 11**	239 ± 31**#
p67^phox^	100 ± 7	144 ± 11	106 ± 9	138 ± 29*
IL-10	100 ± 29	254 ± 72	5776 ± 1633*	7006 ± 2840
**Liver**				
TNF***α***	100 ± 10	41 ± 7**	8560 ± 1284**	21238 ± 4810**##
MCP-1	100 ± 7	53 ± 3**	27787 ± 1580**	29249 ± 2338**
IL-1***β***	100 ± 21	27 ± 3**	6477 ± 691**	7197 ± 1647**
COX-2	100 ± 9	81 ± 24	12335 ± 1128**	6317 ± 707**
gp91^phox^	100 ± 7	119 ± 14	187 ± 13**	261 ± 22**#
p67^phox^	100 ± 9	108 ± 13	1 04 ± 17	129 ± 31
IL-10	100 ± 7	64 ± 10*	11428 ± 1543**	5625 ± 922**

Ten daily doses of ethanol and LPS induced changes in gene expression in brain and liver. Male C57BL/6J mice were treated intragastrically with ethanol (5 g/kg, i.g.) daily for 10 days and injected intraperitoneally (i.p.) with LPS (3 mg/kg) 24 hrs after ethanol treatment. Brain and liver samples were collected 1 hr post LPS treatment. Gene expression of proinflammatory and anti-inflammatory factors was measured by real-time PCR. The results are the means ± SEM (n = 6 per group). * P < 0.05, ** P < 0.01, compared with the saline controls. ^# ^P < 0.05, ^## ^P < 0.01, compared with LPS-treated group.

### TNF*α*, IL-1*β*, MCP-1 and IL-10 assays

Frozen livers and brains were homogenized in 100 mg tissue/ml cold lysis buffer (20 mM Tris, 0.25 M sucrose, 2 mM EDTA, 10 mM EGTA, 1% Triton ×-100) and 1 tablet of Complete Mini protease inhibitor cocktail tablets/10 ml (Roche Diagnostics, Indianapolis, IN). Homogenates were centrifuged at 100,000 × g for 40 min, supernatant was collected, and protein levels determined using the BCA protein assay reagent kit (PIERCE, Milwaukee, WI). The levels of TNFα, MCP-1, IL-1***β ***and IL-10 in livers, sera and brains were measured with TNFα, mouse JE/MCP-1, IL-1***β ***and IL-10 commercial enzyme-linked immunosorbent assay (ELISA) kits from R&D Systems (Minneapolis, MN), as described previously [19].

### Statistical analysis

The data are expressed as mean ± SEM and statistical significance was assessed with an ANOVA followed by Bonferroni's t-test. A value of P < 0.05 was considered statistically significant.

## Results

### Liver, serum and brain pro-inflammatory cytokines

**Figure 1:**
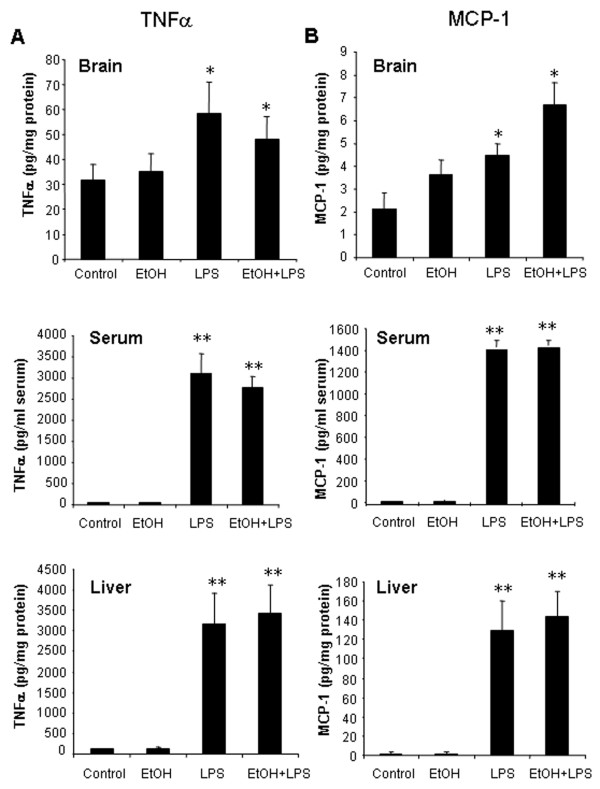
Effects of a single dose of ethanol exposure on LPS-induced liver, serum and brain TNFα production. Male C57BL/6J mice were treated intragastrically with ethanol (5 g/kg, i.g.) for one day. Mice were injected intraperitoneally (i.p.) with LPS (3 mg/kg) 24 hrs after ethanol treatment. Liver, serum and brain samples were collected at 1 hr post LPS treatment. Analysis of TNFα and MCP-1 protein was conducted by ELISA. (A) LPS and ETOH-LPS rapidly increased liver, serum and brain TNFα protein. (B) MCP-1 protein in liver, serum and brain was increased after LPS treatment alone or combined LPS and ethanol treatments. The results are the means ± SEM (n = 6 per group). * P < 0.05, ** P < 0.01, compared with the saline controls.

**Figure 2:**
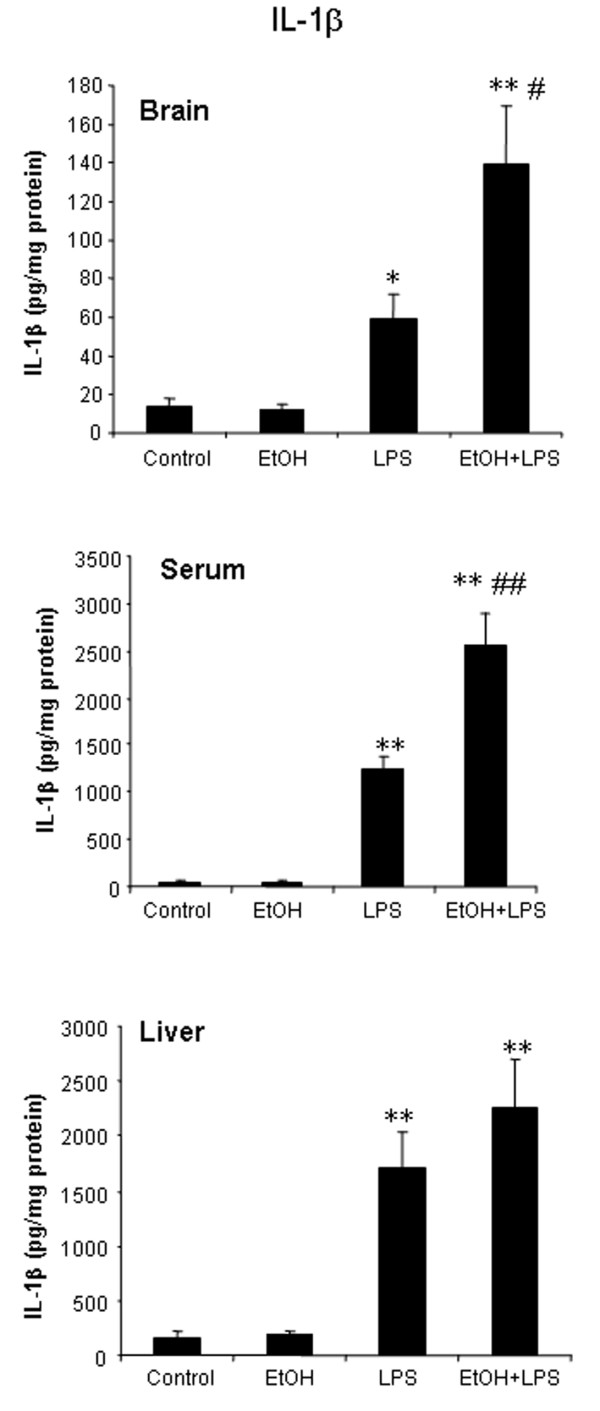
A single dose of ethanol treatment enhanced LPS-induced IL-1***β ***protein in brain and serum, but not in liver. Male C57BL/6J mice were treated intragastrically with ethanol (5 g/kg, i.g.) for one day. Mice were injected intraperitoneally (i.p.) with LPS (3 mg/kg) 24 hrs after ethanol treatment. Liver, serum and brain samples were collected at 1 hr after LPS injection. Liver, serum and brain IL-1***β ***protein was measured by ELISA. (A) A single ethanol dose increased LPS-induced brain IL-1***β ***production. (B) A single dose of ethanol increased LPS-induced IL-1***β ***protein in serum. (C) A single dose of ethanol did not show potentiation to LPS-stimulated increase in IL-1***β ***protein in liver. The results are the means ± SEM (n = 6 per group). * P < 0.05, ** P < 0.01, compared with the saline controls.^# ^P < 0.05, ^## ^P < 0.01, compared with LPS-treated group.

To investigate liver, serum and brain cytokines, endotoxin (lipopolysaccharide, LPS, 3 mg/kg, i.p.) was administered and cytokine levels were determined. LPS induced large increases in TNF***α***, MCP-1, and IL-1***β ***mRNA and protein levels in liver, serum and brain within 1 hour (Fig. 1, 2, Table 2). Changes in mRNA are generally very large and are described as fold increases over control, whereas protein changes tend to be smaller and are presented as percent changes with absolute values in figures and tables. TNF***α ***mRNA was increased 137 fold in liver at 1 hour corresponding to about a 31 fold increase in TNF***α ***protein in liver and 100 fold increase in serum TNF***α ***protein. MCP-1 mRNA was increased 135 fold in liver corresponding to a 54 fold increase in MCP-1 protein in liver and 57 fold increase in serum MCP-1 protein. IL-1β mRNA increased 23 fold in liver corresponding to a 10 fold increase in IL-1β protein in liver and 27 fold increase in serum IL-1β protein. Although the brain also showed marked increases in proinflammatory mRNA and protein the ratios were somewhat different. LPS induced brain TNF***α ***mRNA 47 fold, MCP-1 mRNA 3 fold and IL-1β mRNA 16 fold corresponding to a 2–6 fold increase in protein levels at 1 hour for these proinflammatory cytokines. Although LPS induced increases in TNF***α ***levels in serum and liver peaked at about 1 hour [2], there may be a different time course for other cytokines. Thus, LPS rapidly induces TNF***α***, MCP-1, and IL-1***β ***proinflammatory cytokines in liver, serum and brain.

**Table 2:** A single dose of ethanol and LPS induced changes in gene expression in brain and liver

Gene	Control	ETOH	LPS	ETOH + LPS
**Brain**				
TNF***α***	100 ± 13	413 ± 100*	4595 ± 1360*	19079 ± 6532*
MCP-1	100 ± 5	146 ± 12**	291 ± 65	1294 ± 215*
IL-1***β***	100 ± 15	91 ± 12	1536 ± 385*	4534 ± 810**#
COX-2	100 ± 6	154 ± 13**	386 ± 41**	474 ± 44**
gp91^phox^	100 ± 9	127 ± 9	138 ± 19	228 ± 44*#
p67^phox^	100 ± 10	131 ± 12	116 ± 10	98 ± 18
IL-10	100 ± 13	59 ± 20	1578 ± 825	4864 ± 873*
**Liver**				
TNF***α***	100 ± 7	121 ± 30	13632 ± 2417**	13894 ± 984**
MCP-1	100 ± 9	86 ± 10	13279 ± 2004**	21332 ± 5143**
IL-1***β***	100 ± 15	20 ± 2**	2215 ± 211**	2232 ± 457**
COX-2	100 ± 27	136 ± 11	9663 ± 1056**	8296 ± 467**
gp91^phox^	100 ± 12	62 ± 5*	125 ± 12	131 ± 25
p67^phox^	100 ± 15	61 ± 4*	106 ± 18	81 ± 14
IL-10	100 ± 20	26 ± 4**	3615 ± 394**	4854 ± 979**

Ethanol and LPS induced changes in gene expression in brain and liver. Male C57BL/6J mice were treated intragastrically with ethanol (5 g/kg, i.g.) for one day. Mice were injected intraperitoneally (i.p.) with LPS (3 mg/kg) 24 hrs after ethanol treatment. Brain and liver samples were collected at 1 hr post LPS treatment. Gene expression of proinflammatory and anti-inflammatory factors was measured by real-time PCR. The results are the means ± SEM (n = 6 per group). * P < 0.05, ** P < 0.01, compared with the saline controls. # P < 0.05 compared with LPS-treated group.

To investigate the effects of one dose of ethanol exposure on TNF***α***, MCP-1 and IL-1***β ***in liver, serum, and brain, mice were pretreated with ethanol (5 g/kg, i.g.), a binge drinking dose, and samples were taken 25 hours later to ensure the absence of ethanol. This single dose of ethanol alone did not elevate liver proinflammatory cytokines, whereas brain mRNA for TNF***α***, MCP-1 and COX-2 were increased (Table 2). LPS treatment 24 hours after one dose of ethanol showed a potentiated proinflammatory cytokine response in brain, but not in liver. In brain, the TNF***α ***mRNA response was increased 47 fold by LPS and was potentiated to 196 fold in ethanol-LPS animals (Table 2). Similarly IL-1***β ***mRNA in brain increased 16 fold after LPS and was potentiated to 47 fold by one ethanol pretreatment. Protein levels of TNF***α***, MCP-1, and IL-1***β ***proinflammatory cytokines were increased in all tissues measured. In brain and serum LPS induced IL-1***β ***protein levels were approximately doubled by a single ethanol pretreatment (Fig. 2). One dose of ethanol pretreatment also increased LPS-induced gene expression of gp91^phox^, a subunit of NADPH oxidase, in the brain. Immunohistochemistry for microglia using Iba1 immunostaining in brains of a single ethanol dose and/or LPS treated mice did not show marked morphological indications of activation 1 hr after LPS (images not shown). Interestingly, the mRNA level of IL-10, the regulatory anti-inflammatory cytokine, was increased many fold by LPS in liver and brain, but ethanol pretreatment followed by LPS increased brain IL-10 mRNA response to LPS from 16 to 50 fold, whereas LPS in liver showed 40–50 fold IL-10 mRNA increases due to LPS for IL-10 regardless of ethanol pretreatment. These studies indicate that within 1 hour LPS induces cytokines in liver, serum and brain. ***A ***single dose of ethanol pretreatment potentiates LPS-induced brain cytokine mRNA as well as IL-1***β ***protein in serum and brain.

### Ten daily doses of ethanol induces proinflammatory cytokines and increases cytokine responses to systemic LPS administration

**Figure 3:**
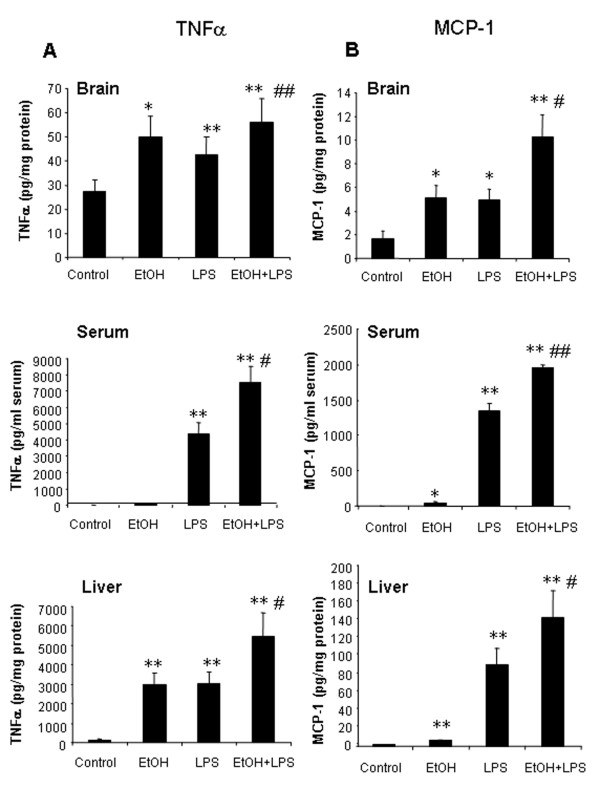
Effects of 10 daily doses of ethanol exposure on LPS-induced liver, serum and brain TNFα and MCP-1 production. Male C57BL/6J mice were treated intragastrically with ethanol (5 g/kg, i.g.) daily for 10 days and injected intraperitoneally (i.p.) with LPS (3 mg/kg) 24 hrs after ethanol treatment. Liver, serum and brain samples were collected 1 hr post LPS treatment. TNFα and MCP-1 protein was determined by ELISA. (A) In 10-day ethanol pre-treated group, LPS significantly increased liver, serum and brain TNFα protein compared with LPS alone group. (B) Exposure to 10 daily doses of ethanol resulted in a significant increase in MCP-1 protein in liver, serum and brain. The results are the means ± SEM of two experiments performed with 6 mice each group. * P < 0.05, ** P < 0.01, compared with the saline controls. ^# ^P < 0.05, ^## ^P < 0.01, compared with LPS-treated group.

**Figure 4:**
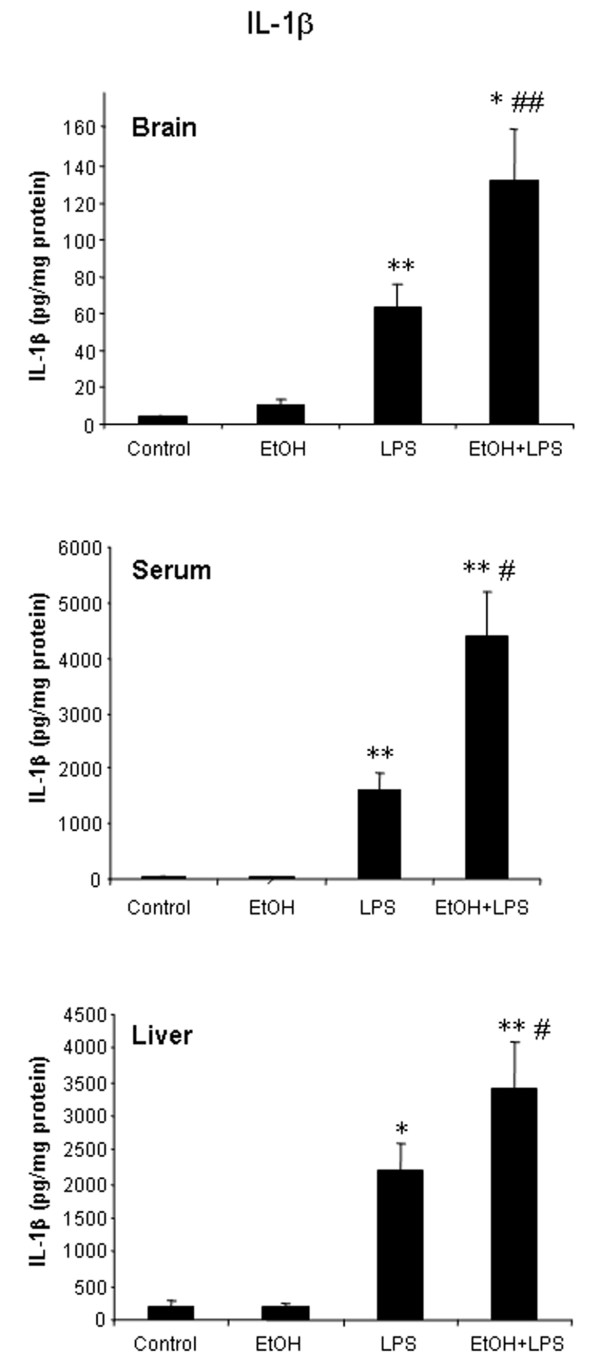
Effects of 10 daily doses of ethanol exposure on LPS-induced liver, serum and brain IL-1***β ***production. Male C57BL/6J mice were treated intragastrically with ethanol (5 g/kg, i.g.) for 10 days and injected intraperitoneally (i.p.) with LPS (3 mg/kg) 24 hrs after ethanol treatment. Liver, serum and brain samples were collected 1 hr after LPS injection. LPS significantly increased IL-1***β ***protein in liver, serum and brain after 10 days of ethanol administration compared with LPS alone treatment. The results are the means ± SEM of two experiments performed with 6 mice each group. * P < 0.05, ** P < 0.01, compared with the saline control mice. ^# ^P < 0.05, ^## ^P < 0.01, compared with LPS-treated mice.

Chronic high alcohol ingestion is common among 20–30% of adults [20]. We administered a heavy drinking dose of ethanol (5 g/kg) daily for 10 days to determine if 10 daily doses of ethanol ingestion with or without LPS would alter cytokines. Interestingly, in 10 daily doses of ethanol treated mice, 25 hrs after the last dose of ethanol, there were significant increases in liver and brain TNF***α ***and MCP-1 protein (Fig. 3). IL-1***β ***did not show an effect with ethanol alone on protein levels in either tissue (Fig. 4). Ten daily doses of ethanol pretreatment significantly enhanced LPS induced TNF***α***, MCP-1 and IL-1***β ***mRNA and protein. Large increases in mRNA were found for TNF***α***, MCP-1 and IL-1***β ***in LPS treated animals, with 10 daily doses of ethanol pretreatment potentiating TNF***α ***mRNA levels in liver one hour after LPS treatment from 87 to 216 fold (Table 1). Ten daily doses of ethanol also increased LPS-induced TNF***β ***protein in liver (178%), serum (172%), and brain (132%), IL-1***β***protein in liver (156%), serum (271%), and brain (210%), and MCP-1 protein in liver (155%), serum (147%), and brain (209%) compared to values for LPS treatment alone. These studies indicate that 10 daily doses of ethanol alone can induce proinflammatory TNF***β ***and MCP-1 protein in brain. Further, 10 daily doses of ethanol pretreatment increases LPS stimulated levels of proinflammatory cytokines in liver, serum and brain.

**Figure 5:**
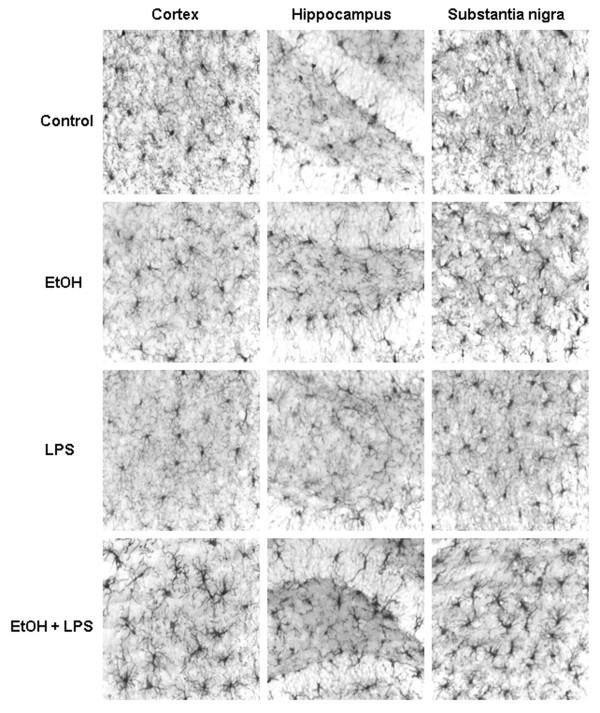
Immunocytochemical analysis of microglia. Male C57BL/6J mice were treated intragastrically with ethanol (5 g/kg, i.g.) for 10 days and injected intraperitoneally (i.p.) with LPS (3 mg/kg, i.p.) 24 hrs after ethanol treatment. Mice were sacrificed 1 hr following saline or LPS injection. Brain sections were fixed and stained with Iba1 antibody. Ten daily doses of ethanol exposure potentiated LPS-induced microglial activation. In either LPS or ethanol treated groups, most of the microglia were in a resting morphological shape. However, in the ethanol pre-treated group, LPS increased Iba1 staining in some brain regions such as cortex, hippocampus and substantia nigra. Iba1-immunoreactive (IR) cells showed an increased cell size, irregular shape consistent with morphological changes in activated microglia.

A number of reports indicate that microglia, the resident innate immune cells in the brain, can become activated in response to diverse stimuli to produce proinflammatory factors such as superoxide, TNF***α***, and IL-1***β ***[6,21,22]. Microglia, identified by Iba1 immunoreactivity (Iba1+IR), were evaluated morphologically for activation. One hour after LPS (3 mg/kg i.p.) or 25 hrs after 10 doses of ethanol (5 g/kg, i.g.) microglia show a resting morphological shape (Fig. 5). However, in the 10 daily doses of ethanol pretreated group, LPS at 1 hour increased Iba1 staining in multiple brain regions, e.g. cortex, hippocampus and substantia nigra. Iba1+IR cells in the 10 daily doses of ethanol-LPS group showed increased cell size and irregular shape consistent with morphological activation of microglia. These studies indicate that 10 daily doses of ethanol increased induction of proinflammatory cytokines and morphological signs of microglial activation by LPS.

**Figure 6:**
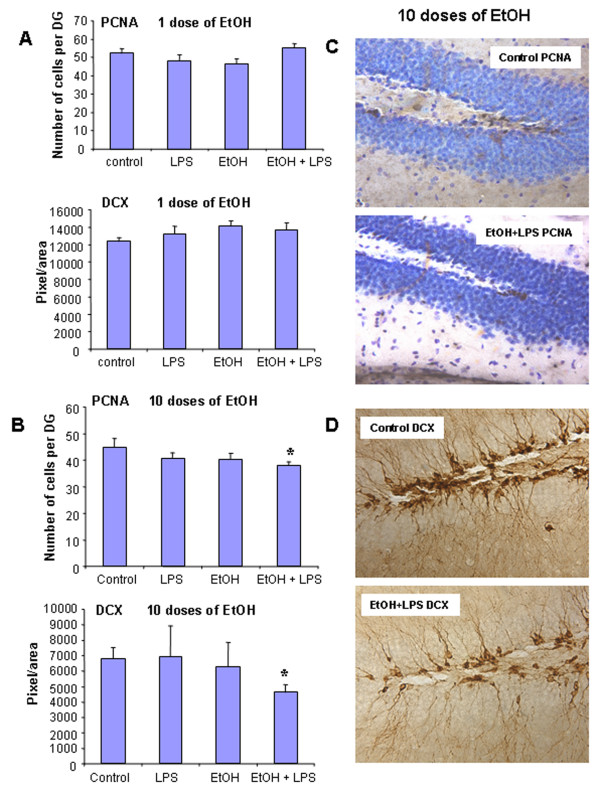
Ten daily doses of ethanol exposure reduced neurogenesis in hippocampal dentate gyrus during combined ethanol and LPS treatments. Male C57BL/6J mice were treated intragastrically with ethanol (5 g/kg, i.g.) for either 1 day or 10 days and injected intraperitoneally (i.p.) with LPS (3 mg/kg, i.p.) 24 hrs after ethanol treatment. Mice were sacrificed 1 hr following saline or LPS administration. Brain sections were fixed and stained with anti-mouse PCNA (a marker for proliferation of neural progenitor cells) and anti-goat doublecortin (a marker for immature or newly born neurons) antibodies. (A) Ethanol (5 g/kg, i.g., 1 day) pre-treated group, LPS did not show a decrease in the number of PCNA and doublecortin-IR cells. (B) Ethanol (5 g/kg, i.g., 10 days) pre-treated mice, LPS significantly decreased PCNA and doublecortin-IR cells, suggesting that 10 daily doses of ethanol inhibits neurogenesis during combined ethanol and LPS treatments. (C) The pictures represent PCNA-IR cells in control (upper panel) and ETOH-LPS treated (lower panel) dentate gyri of the hippocampus. (D) Representative pictures of doublecortin (DCX) immunoreactivity. DCX expression was shown in control brain (upper panel) and ETOH-LPS treated brain (lower panel).

Neuroinflammation is associated with most neurodegenerative diseases. Neurogenesis is a growth and regenerative component of the brain which when inhibited represents a form of degeneration. Neurogenesis is inhibited by chronic LPS induced neuroinflammation [6]. Using PCNA, a marker for neuroprogenitor proliferation, and doublecortin (DCX), a marker for immature or newly born neurons, we assessed neurogenesis in adult hippocampus. In this study, neurogenesis was not decreased 25 hrs after a single ethanol dose or 1 hour after LPS or ethanol-LPS treatments. Neurogenesis was significantly decreased in 10 daily doses of ethanol-LPS treated animals (Fig. 6). Both proliferation of hippocampal neuroprogenitors (PCNA) and new born neuron differentiation (DCX) were decreased in 10 daily doses of ethanol-LPS treated animals that also showed morphology of activated microglia. These findings suggest that the combination of ethanol and LPS insult neurogenesis greater than either treatment alone.

### Persistent long lasting increases in proinflammatory cytokines in brain, but not in liver and serum

**Figure 7:**
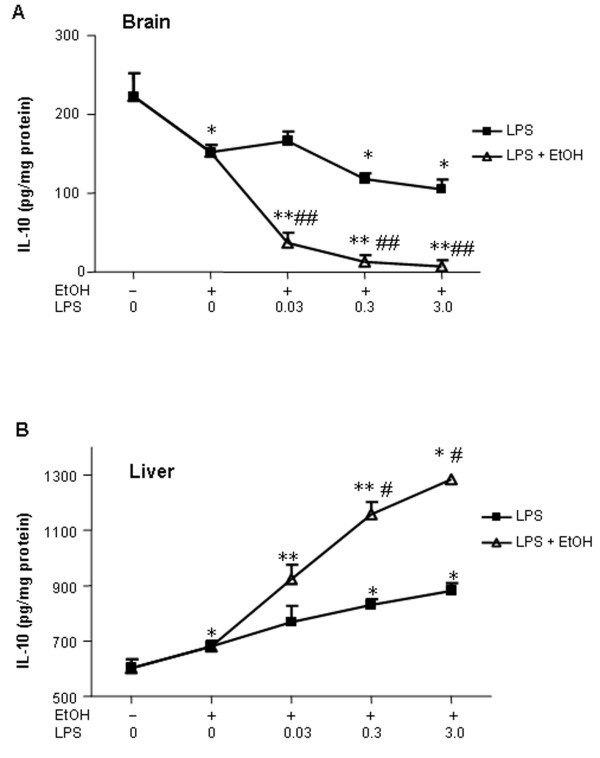
Effects of 10 daily doses of ethanol on IL-10 in brain and liver at 1 week after LPS treatment. Male C57BL/6J mice were treated intragastrically with ethanol (5 g/kg, i.g.) for 10 days and were injected intraperitoneally (i.p.) with LPS at the indicated doses 24 hrs after ethanol treatment. Brains and livers were collected at 1 week after LPS injection. The level of IL-10 protein was measured by ELISA. (A) Ten doses of ethanol decreased brain IL-10 and enhanced LPS-induced decrease in IL-10 in a LPS dose-dependent manner at 1 week after LPS treatment. (B) There was a significant increase in liver IL-10 protein in 10 daily doses of ethanol, LPS (0.3 and 3 mg/kg) and ETOH-LPS groups. P < 0.05, ** P < 0.01, compared with the saline controls.^# ^P < 0.05, ^## ^P < 0.01, compared with the corresponding LPS-treated group.

To investigate the effects of 10 daily doses of ethanol (5 g/kg, i.g.) on the duration of the response, LPS was given 24 hours after the last dose of ethanol, and cytokines were measured 1 week after LPS. In addition, several doses of LPS were used (0.03, 0.3 and 3 mg/kg) in order to better detect the potentiation from 10 daily doses of ethanol treatment. After 1 week, there were no significant differences in liver or serum protein levels of proinflammatory cytokines (TNF***α***, IL-1***β ***and MCP-1) in any groups compared to the saline control group. Similarly liver mRNA for TNF***α***, MCP-1 and IL-1***β ***were at or below control levels 1 week after LPS (Table 3). Interestingly, IL-10 mRNA (Table 3) and protein (Fig. 7) were elevated in liver at this delayed time point, i.e. 8 days after ethanol and/or 7 days after LPS. These findings suggest that the proinflammatory cytokine responses of liver and serum are transient, perhaps due to a delayed increase in IL-10.

**Table 3:** Ten daily doses of ethanol and LPS induced changes in gene expression in brain and liver at 1 week

**Gene**	**Control**	**ETOH**	**LPS 0.03**	**LPS 0.3**	**LPS 3**	**LPS 0.03 + ETOH**	**LPS 0.3 + ETOH**	**LPS 3 + ETOH**
**Brain**								
TNF*β*	100 ± 19	109 ± 17	113 ± 19	78 ± 21*	225 ± 21**	134 ± 6	172 ± 21*	307 ± 47**
MCP-1	100 ± 7	138 ± 5**	109 ± 8	122 ± 5*	163 ± 24*	139 ± 10*	150 ± 5**#	147 ± 13*
IL-1*β*	100 ± 8	114 ± 9	137 ± 10*	132 ± 19	132 ± 13	109 ± 9	108 ± 10	149 ± 23
gp91^phox^	100 ± 12	133 ± 4*	102 ± 14	151 ± 9*	156 ± 17*	166 ± 21*##	182 ± 18**	236 ± 30**#
p67^phox^	100 ± 6	133 ± 12*	113 ± 11	153 ± 6**	164 ± 15**	112 ± 10	136 ± 19	155 ± 22*
DBI	100 ± 16	199 ± 28*	187 ± 15**	181 ± 7**	217 ± 21**	219 ± 7**	239 ± 13**#	188 ± 43
IL-10	100 ± 28	ND	ND	ND	ND	ND	ND	ND
**Liver**								
TNF*β*	100 ± 11	116 ± 11	90 ± 9	110 ± 17	107 ± 16	110 ± 7	95 ± 10	115 ± 11
MCP-1	100 ± 6	110 ± 11	86 ± 16	100 ± 12	87 ± 14	102 ± 4	109 ± 9	104 ± 7
IL-1*β*	100 ± 21	93 ± 19	84 ± 18	63 ± 8	59 ± 6	40 ± 5*	39 ± 3*	43 ± 5*
IL-10	100 ± 21	243 ± 33*	222 ± 25*##	485 ± 92**	212 ± 49	427 ± 29**	312 ± 57*	281 ± 79

ND = not detected. Ten daily doses of ethanol and LPS induced changes in gene expression in brain and liver at 1 week. Male C57BL/6J mice were treated intragastrically with ethanol (5 g/kg, i.g.) daily for 10 days and injected intraperitoneally (i.p.) with LPS (3 mg/kg) 24 hrs after ethanol treatment. Brain and liver samples were collected 1 week post LPS treatment. Gene expression of proinflammatory and anti-inflammatory factors was measured by real-time PCR. The results are the means ± SEM (n = 6 per group). * P < 0.05, ** P < 0.01, compared with the saline controls. ^# ^P < 0.05, ^## ^P < 0.01, compared with LPS-treated group.

**Figure 8:**
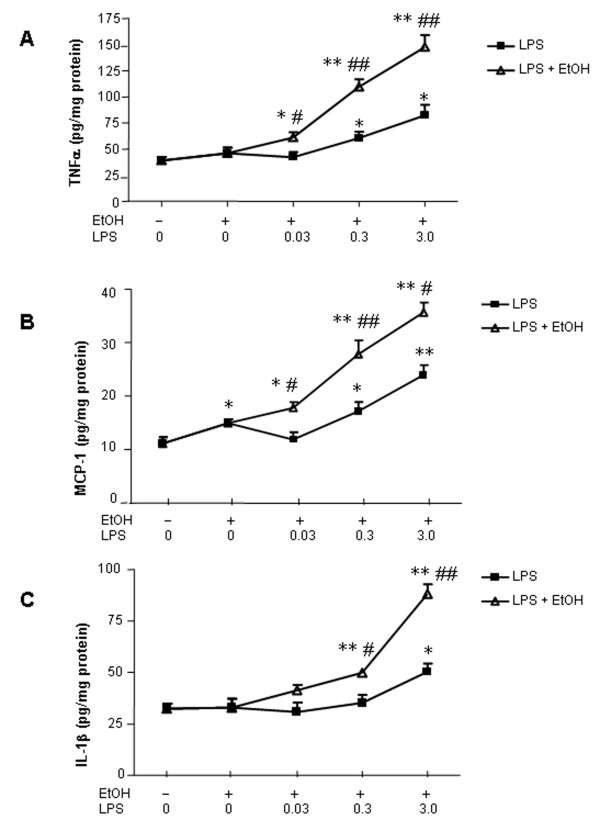
Ten daily doses of ethanol administration potentiated LPS-induced brain TNFα, MCP-1 and IL-1***β ***production that remained elevated at 1 week. Male C57BL/6J mice were treated intragastrically with ethanol (5 g/kg, i.g.) for 10 days and were injected intraperitoneally (i.p.) with LPS at indicated doses 24 hrs after ethanol treatment. Brains were collected at 1 week after LPS injection. The levels of TNFα, MCP-1 and IL-1***β***protein were measured by ELISA. (A) In the10-dose ethanol pre-treated group, brain TNF***β ***protein significantly increased in a LPS dose-dependent manner. (B) Exposure to 10 daily doses of ethanol resulted in a significant increase in brain MCP-1 in a LPS dose-dependent manner. (C) Brain IL-1***β***protein synthesis was also enhanced by ethanol pre-treatment. The results are the means ± SEM (n = 6 per group). * P < 0.05, ** P < 0.01, compared with the saline controls.^#^P < 0.05, ^## ^P < 0.01, compared with the corresponding LPS-treated group.

Although induction of proinflammatory cytokines in liver and serum were transient, brain showed persistent elevation of proinflammatory genes by both LPS and ethanol. Remarkably, in brain MCP-1 protein and mRNA were increased (138%, p < 0.05 for protein; 1.38 fold, p < 0.01 for mRNA) 8 days after the last ethanol treatment (Fig. 8, Table 3). Brain MCP-1 mRNA was increased 24 hrs after a single dose of ethanol (Table 2) and both protein and mRNA were increased after 10 daily doses of ethanol (Table 1, Fig. 3) and remained increased 8 days after 10 daily doses of ethanol exposure (Table 3, Fig. 8) indicating that this proinflammatory cytokine is exceptionally responsive to ethanol. The proinflammatory enzyme that makes reactive oxygen species, NADPH oxidase, showed increased mRNA for subunits, gp91^phox^, and p67^phox ^that persist for 1 week after ethanol and/or LPS.

Also elevated in brain 1 week after ethanol and/or LPS was diazepam binding inhibitor (DBI), a regulator of microglial steroid metabolism induced by proinflammatory stimuli (Table 3). Ten daily doses of ethanol pretreatment increased both the potency and efficacy of LPS at inducing both protein and mRNA levels of ***TN***F***α***, MCP-1, and IL-1***β ***(Fig. 8, Table 3) and markers of neuroinflammation e.g. mRNA for diazepam binding inhibitor (DBI), and NADPH oxidase (Table 3). Although proinflammatory cytokines and other markers of inflammation remained elevated, markers of neurogenesis, e.g. PCNA and DCX, had returned to control levels 1 week after LPS with or without ethanol pretreatment (data not shown). Thus, increases in brain proinflammatory cytokines and genes persist, although liver and serum proinflammatory cytokines return to control levels after 1 week.

The differential response of liver and serum as compared to the brain could be related to differences between brain and liver IL-10, an anti-inflammatory cytokine. Ten daily doses of ethanol significantly reduced brain IL-10 production and enhanced a decrease in LPS-induced brain IL-10 protein in a LPS dose-dependent manner (Fig. 7). Interestingly, the liver was opposite to that of the brain, with both ethanol and LPS causing a significant increase in liver IL-10 protein. Ten daily doses of ethanol pretreatment potentiated both the LPS induced decrease in IL-10 in brain and the LPS-induced increase in IL-10 in liver (Fig. 7). Thus, in brain, 10 daily doses of ethanol induced a long lasting increase in proinflammatory cytokines and potentiated multiple brain LPS proinflammatory cytokine responses while decreasing the anti-inflammatory cytokine IL-10. In the liver and serum, ethanol induced proinflammatory cytokines and increased multiple brain LPS proinflammatory cytokine responses at 1 hr, the peak of the TNF***α ***response, whereas hepatic and systemic levels of proinflammatory cytokines subsided after 1 week when the anti-inflammatory cytokine IL-10 was increased.

## Discussion

To our knowledge, this is the first report to show the striking concordance of proinflammatory cytokine (TNFα, MCP-1, and IL-1β) responses in liver, serum and brain to LPS with or without ethanol pretreatment. The fascinating discovery of the correlation of responses in liver, serum and brain at 1 hour after LPS and/or ethanol, are likely due to liver and serum monocyte-macrophage like cells secreting cytokines into serum that are transported into brain. *In vivo*, normal liver secretes little or no TNF***α***, however, in response to LPS liver Kupffer cells, resident hepatic macrophages, secrete TNF***β ***and other cytokines [23]. Kupffer cell removal from the liver blunts LPS-induced proinflammatory cytokines (TNF***α ***and IL-1β) in serum, lung and kidney [24]. These data suggest that liver Kupffer cells are responsible for a significant portion of TNFα production in serum, which in turn could trigger more TNF***α ***in other tissues. Blood-born monocytes likely contribute to serum levels as well. Transport across the blood brain barrier has been reported for many cytokines including TNF and IL-1, but not IL-10 [25]. Studies using ^125^I-TNF***α ***in mice have found that TNFα is transported into the brain within minutes after an intravenous injection [26], likely by TNF receptors used as transporters. Previously, we found that in TNF receptor 1 and 2 double knock out mice (TNFR1/R2-KO) did not show significant brain induction of proinflammatory cytokines (TNF***α***, MCP-1) after injections of TNFα or LPS, whereas wild type mice did show brain induction of proinflammatory cytokines after peripheral injection of TNFα or LPS [2]. These findings are consistent with TNFα and other proinflammatory cytokines in serum carrying the proinflammatory response from the serum into the brain. Thus, the concordance of liver, serum and brain induced proinflammatory cytokine responses at 1 hour after LPS injection with or without ethanol pretreatment is likely due to cytokine synthesis from both liver Kupffer and blood monocyte cells resulting in increasing serum levels that are transported into brain inducing brain production of proinflammatory cytokines.

Proinflammatory cytokine induction in liver and serum subsides by one week. Surprisingly, IL-10, an anti-inflammatory cytokine, was induced by LPS at short time points similar to proinflammatory cytokines; however, the IL-10 response persisted in liver whereas the proinflammatory cytokines returned to control level. In human blood monocytes IL-10 inhibits LPS and TNF***α ***induction of IL-1***β ***and TNF***α ***by blocking NF-***κ ***B transcription [27,28]. In liver, cytokines regulate immune response progression from the early proinflammatory state that enhances antigen presentation and Th1 lymphocyte infiltration to the anti-inflammatory Th2 lymphocyte state [1]. Our studies suggest that liver and systemic proinflammatory cytokines are suppressed by IL-10, perhaps from regulatory lymphocytes, whereas the decreased IL-10 in the brain where there are normally no lymphocytes, may contribute to the prolonged increase in proinflammatory cytokines. It is possible that ethanol induction of anti-inflammatory IL-10 could have health benefit for diseases associated with 10 daily doses of inflammation and autoimmunity, however, additional studies need to be done to understand the differential response of cytokines in the brain versus the liver and serum.

Previous studies investigating the effects of ethanol in vivo (i.g.) on serum LPS responses found that a single dose of ethanol given before LPS can inhibit serum IL-6 and serum acute phase response proteins indicative of inflammation [29]. Similar to our study, a binge drinking dose of ethanol alone increased serum acute phase proteins with a peak response around 24 hrs. Low doses of ethanol before LPS suppressed the LPS serum response. Our findings that 10 daily binge doses of ethanol pretreatment enhances the LPS-induced TNF***α***, MCP-1, IL-1***β ***and NADPH oxidase gp91^phox ^subunit responses to LPS may represent a type of supersensitivity. Ethanol is an antagonist when it is present that results in an increase in sensitivity that lasts longer than the blood ethanol level. Human monocytes isolated from the blood of alcoholics are known to produce greater amounts of TNFα spontaneously and in response to endotoxin challenge [13]. Similarly, liver Kupffer cell cultures isolated from chronic ethanol pretreated rats have increased LPS-induced calcium flux and increased production of TNF***α ***[30] due in part to ethanol up-regulation of the LPS receptor CD14 [31,32]. Thus, ethanol potentiation of short term liver and serum induction of proinflammatory cytokines is likely due to increased liver Kupffer cell macrophage and blood monocyte secretion of TNF***α ***following chronic ethanol pretreatment.

Ethanol pretreatment followed by LPS increased brain levels of proinflammatory cytokines that persist for at least one week. We previously found that a single dose of LPS (5 mg/kg, i.p.) can induce brain TNF***α ***for up to 10 months [2]. We report here that both LPS and ethanol induce long lasting increases in several brain proinflammatory cytokines (LPS: TNF***α***, MCP-1, IL-1***β ***and ethanol: TNF***α***, MCP-1) and cause long lasting reductions in brain anti-inflammatory cytokine IL-10. Ethanol not only sensitizes proinflammatory cytokine production, but also increases morphological signs of microglial response, e.g. increased cell size, irregular shape and intensified Iba1 staining. Neurogenesis represents a growth and regenerative component of adult brain which when inhibited represents a form of degeneration. In this study, we found that neurogenesis was significantly decreased in 10 daily doses of ethanol-LPS treated animals. Both proliferation of hippocampal neuroprogenitors and newly born neuron differentiation were significantly decreased in 10 daily doses of ethanol-LPS treated animals. Multiple doses of LPS reduce neurogenesis that is reversed by indomethicin, a common anti-inflammatory drug [6], suggesting that inflammation can inhibit neurogenesis. It is possible that ethanol enhancement of LPS induced proinflammatory cytokines results in greater inflammation that contributes to the inhibition of neurogenesis. Neurodegeneration is associated with inflammation and oxidative stress [33,34]. Whether inflammation may cause oxidative stress in systemic LPS and alcohol treated animals is unknown. Here we report that 10 daily doses of ethanol significantly up-regulates NADPH oxidase (the main ROS-producing enzyme during inflammation) subunits, gp91^phox ^and p67^phox^, and potentiates LPS-induced induction of these two subunits that persists for at least 1 week (Table 1 and 3). These changes coincide with microglial activation and production of proinflammatory cytokines observed in either LPS or ETOH-LPS treated groups. LPS and ethanol induced persistent proinflammatory cytokine induction could impact neurodegenerative diseases long after systemic infection and alcohol induced systemic proinflammatory cytokines.

Disruption of cytokine cascades has been implicated in a variety of diseases including cardiovascular, liver, respiratory, inflammatory, metabolic, and brain diseases as well as certain cancers. Our findings of altered systemic cytokine levels following ethanol may have broad effects on health. In humans chronic alcohol consumption is associated with increases in serum proinflammatory cytokines [11,12] and monocytes isolated from the blood of alcoholics produce greater amounts of TNFα spontaneously and in response to endotoxin [13]. Similarly, chronic alcohol drinking in female rats results in increased levels of ovarian, pituitary and hypothalamic TNFα and IL-6 [35], likely due to systemic proinflammatory cytokine activation. It is possible that the alcohol induced increased risk for cardiovascular, liver, respiratory, infectious, mental and neurological diseases as well as certain cancers [7] is related to alcohol disruption of cytokine cascades. Our discovery that ethanol increases systemic induction of TNF***α***, MCP-1 and IL-1***β***, and increases NADPH oxidase could contribute to pathologies in multiple tissues and organ systems. Our finding that the systemic potentiation initiates a long lasting self-propelling brain proinflammatory cytokine cascade including up-regulation of NADPH oxidase could contribute to the multiple CNS pathologies including alcoholic dementia. Additional studies will be needed to understand the role of systemic proinflammatory cytokines in chronic diseases and how prolonged increases in proinflammatory cytokines in brain contribute to neurodegeneration and other CNS pathologies.

## Conclusion

In summary, we report that LPS increases liver, serum and brain proinflammatory cytokines. One dose of ethanol induced TNFα, MCP-1 and COX-2 proinflammatory mRNA in brain, but not liver. Pretreatment with a single dose of ethanol potentiated LPS induction of proinflammatory mRNA in brain for NADPH oxidase gp91^phox ^subunit as well as increasing both IL-1β mRNA and protein in brain. Treatment with ethanol for 10 days increased proinflammatory cytokines, TNF***α***, MCP-1 mRNA and protein in brain as well as mRNA for NADPH oxidase gp91^phox^subunit. Pretreatment for 10 days with ethanol potentiated LPS-induced proinflammatory cytokines in liver, serum and brain, as well as the production of anti-inflammatory cytokine, IL-10, in liver, but not in brain. Brain proinflammatory cytokines and enzymes remain elevated after liver and serum proinflammatory cytokines have returned to control levels. Prolonged increases in proinflammatory cytokines may contribute to a variety of systemic and central nervous system pathologies.

## Abbreviations

LPS: Lipopolysaccharide; PCR: Polymerase chain reaction; TNF***α****:*Tumor necrosis factor-***α***; IL-1***β***:****Interleukin-1***β***; IL-10: Interleukin-10; IL-6: Interleukin-6; MCP-1: Monocyte chemotactic protein-1; ELISA: Enzyme-Linked ImmunoSorbent Assay; COX-2: Cyclooxygenase-2; TLR4: The toll-like receptor 4; HIV: Human immunodeficiency virus; PCNA: The proliferating cell nuclear antigen; DCX: Doublecortin; DBI: Diazepam binging inhibitor; CNS: Central nervous system; IACUC: Immunoreactive (IR) and Institutional Animal Care and Use Committee.

## Competing interests

The authors declare that they have no competing interests.

## Authors' contributions

LQ contributed to experimental design, and performed animal experiments, ELISA, immunohistochemistry, data analysis and manuscript preparation. JH performed animal experiments, performed immunohistochemistry of neurogenesis and analyzed data. RNH prepared RNA from tissue samples, reverse transcription, optimized primer sets, performed real-time PCR, analyzed data, and revised the manuscript. OP conducted animal experiments and tissue sample collection. JSH contributed to study design and manuscript revision. FTC designed this study, analyzed data, drafted the manuscript and obtained funding for these studies. All authors read and approved the final manuscript.
